# Hexaaqua­cobalt(II) 3,3′-dicarb­oxy­biphenyl-4,4′-dicarboxyl­ate

**DOI:** 10.1107/S1600536810028771

**Published:** 2010-07-24

**Authors:** Yu-Hua Zhang, Jin-Mei Han, Zong-Ze Li

**Affiliations:** a School of Chemical Engineering and Technology, China University of Mining and Technology, Xuzhou, Jiangsu 221008, People’s Republic of China; b School of Chemical Science and Technology, Key Laboratory of Medicinal Chemistry for Natural Resource, Ministry of Education, Yunnan University, Kunming 650091, People’s Republic of China

## Abstract

In the crystal structure of the title compound, [Co(H_2_O)_6_](C_16_H_8_O_8_), both the cation and anion are centrosymmetric. The Co cation displays a CoO_6_ octa­hedral geometry formed by six water mol­ecules. In the anion, the two carboxyl groups are oriented at dihedral angles of 4.8 (5) and 10.4 (7)° with respect to the benzene ring. Very strong O—H⋯O hydrogen bonds between the protonated and deprotonated carboxylate groups occur. Neighbouring cations and anions are connected through O—H⋯O hydrogen bonds into a three-dimensional supra­molecular structure.

## Related literature

For related metal complexes with the biphenyl-3,3′,4,4′-tetra­carboxyl­ate ligand, see: Sun *et al.* (2009 ▶); Wang *et al.* (2005 ▶, 2006 ▶). For the structures containing the 4,4′-dicarb­oxy­biphenyl-3,3′-dicarboxyl­ate ligand, see: Kang *et al.* (2009*a* ▶,*b* ▶); Zhu *et al.* (2008 ▶).
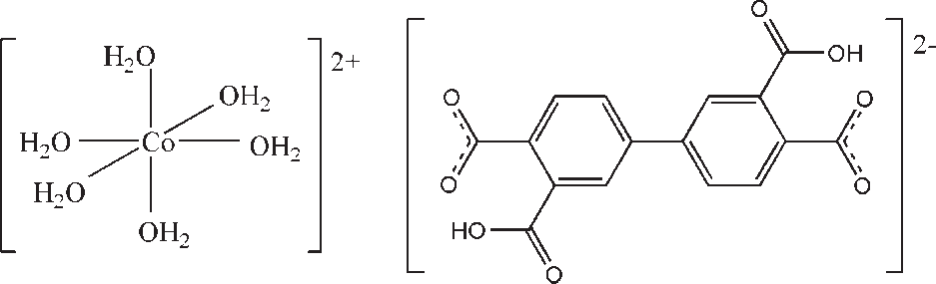


## Experimental

### 

#### Crystal data

[Co(H_2_O)_6_](C_16_H_8_O_8_)
*M* *_r_* = 495.25
Triclinic, 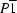

*a* = 6.5197 (14) Å
*b* = 7.9514 (17) Å
*c* = 9.664 (2) Å
α = 76.339 (2)°
β = 87.656 (2)°
γ = 86.221 (2)°
*V* = 485.57 (18) Å^3^
*Z* = 1
Mo *K*α radiation
μ = 0.96 mm^−1^
*T* = 293 K
0.23 × 0.19 × 0.12 mm

#### Data collection

Bruker APEXII CCD area-detector diffractometer
Absorption correction: multi-scan (*SADABS*; Bruker, 2001 ▶) *T*_min_ = 0.804, *T*_max_ = 0.895
2871 measured reflections
1590 independent reflections
1305 reflections with *I* > 2σ(*I*)
*R*_int_ = 0.028

#### Refinement

*R*[*F* ^2^ > 2σ(*F* ^2^)] = 0.048
*wR*(*F* ^2^) = 0.169
*S* = 1.00
1590 reflections
146 parameters
1 restraint
H atoms treated by a mixture of independent and constrained refinement
Δρ_max_ = 0.47 e Å^−3^
Δρ_min_ = −0.43 e Å^−3^

### 

Data collection: *APEX2* (Bruker, 2007 ▶); cell refinement: *SAINT* (Bruker, 2007 ▶); data reduction: *SAINT*; program(s) used to solve structure: *SHELXTL* (Sheldrick, 2008 ▶); program(s) used to refine structure: *SHELXTL*; molecular graphics: *SHELXTL*; software used to prepare material for publication: *SHELXTL*.

## Supplementary Material

Crystal structure: contains datablocks I, global. DOI: 10.1107/S1600536810028771/xu2789sup1.cif

Structure factors: contains datablocks I. DOI: 10.1107/S1600536810028771/xu2789Isup2.hkl

Additional supplementary materials:  crystallographic information; 3D view; checkCIF report

## Figures and Tables

**Table 1:** Selected bond lengths (Å)

Co1—O5	2.054 (3)
Co1—O6	2.027 (3)
Co1—O7	2.082 (3)

**Table 2:** Hydrogen-bond geometry (Å, °)

*D*—H⋯*A*	*D*—H	H⋯*A*	*D*⋯*A*	*D*—H⋯*A*
O2—H2⋯O4	0.85 (2)	1.55 (2)	2.391 (5)	173 (8)
O5—H5*B*⋯O4^i^	0.96	2.17	2.820 (5)	124
O5—H5*C*⋯O2^ii^	0.96	1.97	2.789 (4)	142
O6—H6*A*⋯O3^iii^	0.96	1.84	2.676 (4)	144
O6—H6*C*⋯O1^ii^	0.96	1.79	2.708 (4)	159
O7—H7*A*⋯O1^iv^	0.96	1.83	2.749 (5)	159
O7—H7*C*⋯O3	0.96	1.99	2.822 (5)	144

Symmetry codes: (i) 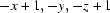; (ii) 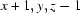; (iii) 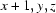; (iv) 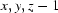.

**Fig. 1.**
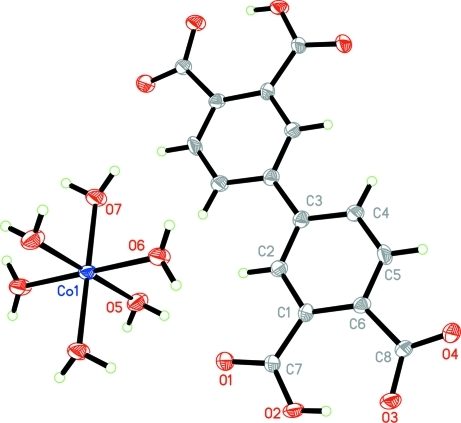
The molecular structure of the title complex with the atom-numbering diagram. Ellipsoids were drawn at the 30% probability level.

**Fig. 2.**
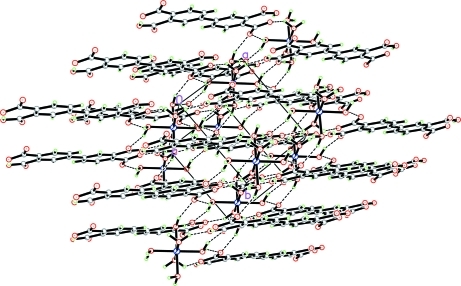
The packing diagram of (I). Hydrogen bonds are marked by dashed line.

## supplementary crystallographic information

### Comment

Biphenyl–3,3',4,4'–tetracarboxylic acid have been used to construct high–dimensional supramolecular networks due to their versatile coordination modes and potential covalent or hydrogen bonding interactions with related parts in the assembly process (Sun *et al.* 2009; Wang *et al.* 2005) such as one-dimensional covalent zigzag chain coexist with one-dimensional hydrogen-bonded ladder (Wang *et al.* 2006). Here we reported a mononuclear complex, containing two ionic components of complex [Co(H_2_O)_6_](C_16_H_18_O_8_) (I) in which the two parts are connected *via* O—H···O hydrogen bonds forming a three-dimensional framework. The structure of the compound (I) consists of discrete ionic entities. A labeled diagram of the crystal [Co(H_2_O)_6_](C_16_H_18_O_18_) is shown in Fig. 1. In the cations, the metal atom is surrounded by six aqua ligands, exhibiting a slightly distorted octahedral stereochemistry. The *cis*/*trans* O—Co—O angles are nearly 90 °. The average Co—O distance for compound (I) is 2.077 Å. The anion 3,3',4,4'–biphenyltetralate contain inversion center. The mean plane was calculated throughout the six atoms of the benzene ring. Because of symmetric reason, the two benzene rings of the biphenyl ligand are coplanar. The carboxylate groups are almost coplanar with the benzene ring with the largest deviation of -0.205 (6) Å for O4. As expected, there are considerable hydrogen bonds in the structure. The bond distances and angles are listed in Table 2. A three–dimensional structure was formed *via* three kinds of hydrogen bonds between the coordinated water molecules and carboxyl groups which also help to consolide the crystal packing (Fig. 2).

### Experimental

A mixture of biphenyl-3,3',4,4'-tetracarboxylic acid (0.2 mmol) and Co(NO_3_)_2_.6H_2_O (0.4 mmol) in 12 ml methanol/water (8:3) sealed in a 25 ml Telflon-lined stainless steel autoclave was kept at 393 K for three days. Single crystals suitable for the X-ray experiment were obtained.

### Refinement

The carboxyl H atom was located in a difference map and refined isotropically. The H atoms of aromatic ring and water molecules were generated geometrically and were included in the refinement in the riding model approximation with C—H = 0.93 Å, U_iso_(H)= 1.2 U_eq_(C) and O—H = 0.96 Å, U_iso_(H)= 1.5U_eq_(O).

### Crystal data

**Table:**

[Co(H_2_O)_6_](C_16_H_8_O_8_)	*Z* = 1
*M**_r_* = 495.25	*F*(000) = 255
Triclinic, *P*1	*D*_x_ = 1.694 Mg m^−^^3^
*a* = 6.5197 (14) Å	Mo *K*α radiation, λ = 0.71073 Å
*b* = 7.9514 (17) Å	Cell parameters from 774 reflections
*c* = 9.664 (2) Å	θ = 2.2–25.0°
α = 76.339 (2)°	µ = 0.96 mm^−^^1^
β = 87.656 (2)°	*T* = 293 K
γ = 86.221 (2)°	Block, pink
*V* = 485.57 (18) Å^3^	0.23 × 0.19 × 0.12 mm

### Data collection

**Table:**

Bruker APEXII CCD area-detector diffractometer	1590 independent reflections
Radiation source: fine-focus sealed tube	1305 reflections with *I* > 2σ(*I*)
graphite	*R*_int_ = 0.028
φ and ω scans	θ_max_ = 25.0°, θ_min_ = 2.2°
Absorption correction: multi-scan (*SADABS*; Bruker, 2001)	*h* = −7→7
*T*_min_ = 0.804, *T*_max_ = 0.895	*k* = −9→9
2871 measured reflections	*l* = −11→10

### Refinement

**Table:**

Refinement on *F*^2^	Primary atom site location: structure-invariant direct methods
Least-squares matrix: full	Secondary atom site location: difference Fourier map
*R*[*F*^2^ > 2σ(*F*^2^)] = 0.048	Hydrogen site location: inferred from neighbouring sites
*wR*(*F*^2^) = 0.169	H atoms treated by a mixture of independent and constrained refinement
*S* = 1.00	*w* = 1/[σ^2^(*F*_o_^2^) + (0.1263*P*)^2^] where *P* = (*F*_o_^2^ + 2*F*_c_^2^)/3
1590 reflections	(Δ/σ)_max_ < 0.001
146 parameters	Δρ_max_ = 0.47 e Å^−^^3^
1 restraint	Δρ_min_ = −0.43 e Å^−^^3^

### Special details

**Table:**

Geometry. All e.s.d.'s (except the e.s.d. in the dihedral angle between two l.s. planes) are estimated using the full covariance matrix. The cell e.s.d.'s are taken into account individually in the estimation of e.s.d.'s in distances, angles and torsion angles; correlations between e.s.d.'s in cell parameters are only used when they are defined by crystal symmetry. An approximate (isotropic) treatment of cell e.s.d.'s is used for estimating e.s.d.'s involving l.s. planes.
Refinement. Refinement of *F*^2^ against ALL reflections. The weighted *R*-factor *wR* and goodness of fit *S* are based on *F*^2^, conventional *R*-factors *R* are based on *F*, with *F* set to zero for negative *F*^2^. The threshold expression of *F*^2^ > σ(*F*^2^) is used only for calculating *R*-factors(gt) *etc*. and is not relevant to the choice of reflections for refinement. *R*-factors based on *F*^2^ are statistically about twice as large as those based on *F*, and *R*- factors based on ALL data will be even larger.

### Fractional atomic coordinates and isotropic or equivalent isotropic displacement parameters (Å^2^)

**Table:**

	*x*	*y*	*z*	*U*_iso_*/*U*_eq_	
Co1	1.0000	0.0000	0.5000	0.0281 (3)	
O1	0.5091 (5)	0.1808 (4)	1.2457 (3)	0.0408 (8)	
O2	0.3077 (5)	0.1278 (4)	1.0880 (4)	0.0396 (8)	
O3	0.4682 (5)	0.2634 (5)	0.6417 (4)	0.0494 (10)	
O4	0.2791 (5)	0.1755 (4)	0.8356 (3)	0.0417 (8)	
O5	1.0135 (5)	−0.0174 (5)	0.2911 (3)	0.0434 (9)	
H5B	0.9751	−0.1302	0.2858	0.065*	
H5C	1.1512	−0.0002	0.2537	0.065*	
O6	1.2248 (5)	0.1707 (5)	0.4594 (4)	0.0444 (9)	
H6A	1.3079	0.1541	0.5419	0.067*	
H6C	1.3089	0.1508	0.3803	0.067*	
O7	0.7843 (5)	0.2081 (4)	0.4461 (4)	0.0441 (9)	
H7A	0.6763	0.1760	0.3946	0.066*	
H7C	0.7277	0.2372	0.5314	0.066*	
C1	0.6112 (6)	0.2899 (5)	1.0027 (4)	0.0255 (9)	
C2	0.7719 (6)	0.3637 (5)	1.0551 (5)	0.0257 (9)	
H2B	0.7823	0.3463	1.1532	0.031*	
C3	0.9157 (6)	0.4613 (5)	0.9679 (5)	0.0267 (9)	
C4	0.8945 (7)	0.4888 (6)	0.8216 (5)	0.0336 (10)	
H4B	0.9850	0.5580	0.7599	0.040*	
C5	0.7413 (7)	0.4147 (6)	0.7674 (5)	0.0358 (11)	
H5A	0.7335	0.4324	0.6691	0.043*	
C6	0.5974 (6)	0.3144 (5)	0.8535 (5)	0.0279 (9)	
C7	0.4682 (6)	0.1923 (5)	1.1202 (5)	0.0288 (10)	
C8	0.4405 (7)	0.2462 (6)	0.7705 (5)	0.0311 (10)	
H2	0.293 (12)	0.152 (9)	0.999 (2)	0.10 (3)*	

### Atomic displacement parameters (Å^2^)

**Table:**

	*U*^11^	*U*^22^	*U*^33^	*U*^12^	*U*^13^	*U*^23^
Co1	0.0215 (5)	0.0511 (6)	0.0143 (5)	−0.0164 (3)	0.0002 (3)	−0.0092 (4)
O1	0.0359 (17)	0.070 (2)	0.0193 (19)	−0.0237 (16)	0.0020 (14)	−0.0103 (15)
O2	0.0344 (17)	0.062 (2)	0.025 (2)	−0.0271 (15)	0.0024 (15)	−0.0103 (16)
O3	0.046 (2)	0.087 (3)	0.024 (2)	−0.0326 (19)	0.0002 (16)	−0.0224 (17)
O4	0.0316 (17)	0.066 (2)	0.032 (2)	−0.0238 (15)	0.0005 (14)	−0.0133 (16)
O5	0.0368 (18)	0.077 (2)	0.0222 (18)	−0.0290 (16)	0.0048 (14)	−0.0167 (16)
O6	0.0373 (18)	0.074 (2)	0.028 (2)	−0.0310 (16)	0.0045 (15)	−0.0191 (16)
O7	0.0361 (18)	0.070 (2)	0.0301 (19)	−0.0101 (16)	−0.0049 (15)	−0.0162 (16)
C1	0.025 (2)	0.032 (2)	0.022 (2)	−0.0069 (16)	0.0007 (17)	−0.0092 (17)
C2	0.026 (2)	0.034 (2)	0.018 (2)	−0.0103 (17)	−0.0028 (17)	−0.0074 (17)
C3	0.024 (2)	0.033 (2)	0.024 (2)	−0.0054 (17)	−0.0038 (18)	−0.0064 (17)
C4	0.038 (2)	0.045 (2)	0.022 (2)	−0.023 (2)	−0.0027 (19)	−0.0090 (19)
C5	0.042 (3)	0.045 (3)	0.024 (3)	−0.019 (2)	−0.002 (2)	−0.0117 (19)
C6	0.024 (2)	0.034 (2)	0.029 (3)	−0.0078 (17)	−0.0020 (18)	−0.0103 (18)
C7	0.028 (2)	0.037 (2)	0.026 (3)	−0.0109 (18)	0.0014 (19)	−0.0126 (18)
C8	0.029 (2)	0.040 (2)	0.029 (3)	−0.0118 (19)	−0.0047 (19)	−0.0137 (19)

### Geometric parameters (Å, °)

**Table:**

Co1—O5	2.054 (3)	O7—H7A	0.9600
Co1—O5^i^	2.054 (3)	O7—H7C	0.9600
Co1—O6	2.027 (3)	C1—C2	1.401 (6)
Co1—O6^i^	2.027 (3)	C1—C6	1.415 (6)
Co1—O7	2.082 (3)	C1—C7	1.534 (6)
Co1—O7^i^	2.082 (3)	C2—C3	1.383 (6)
O1—C7	1.233 (5)	C2—H2B	0.9300
O2—C7	1.276 (5)	C3—C4	1.390 (6)
O2—H2	0.85 (2)	C3—C3^ii^	1.516 (8)
O3—C8	1.226 (6)	C4—C5	1.374 (6)
O4—C8	1.295 (5)	C4—H4B	0.9300
O5—H5B	0.9600	C5—C6	1.389 (6)
O5—H5C	0.9601	C5—H5A	0.9300
O6—H6A	0.9600	C6—C8	1.526 (6)
O6—H6C	0.9600		
			
O6—Co1—O6^i^	180.0	C2—C1—C6	118.3 (4)
O6—Co1—O5	90.40 (13)	C2—C1—C7	113.4 (4)
O6^i^—Co1—O5	89.60 (13)	C6—C1—C7	128.3 (4)
O6—Co1—O5^i^	89.60 (13)	C3—C2—C1	123.2 (4)
O6^i^—Co1—O5^i^	90.40 (13)	C3—C2—H2B	118.4
O5—Co1—O5^i^	180.0	C1—C2—H2B	118.4
O6—Co1—O7	88.72 (14)	C2—C3—C4	117.5 (4)
O6^i^—Co1—O7	91.28 (14)	C2—C3—C3^ii^	120.3 (5)
O5—Co1—O7	89.16 (14)	C4—C3—C3^ii^	122.2 (5)
O5^i^—Co1—O7	90.84 (14)	C5—C4—C3	120.6 (4)
O6—Co1—O7^i^	91.28 (14)	C5—C4—H4B	119.7
O6^i^—Co1—O7^i^	88.72 (14)	C3—C4—H4B	119.7
O5—Co1—O7^i^	90.83 (14)	C4—C5—C6	122.6 (4)
O5^i^—Co1—O7^i^	89.17 (14)	C4—C5—H5A	118.7
O7—Co1—O7^i^	180.000 (1)	C6—C5—H5A	118.7
C7—O2—H2	111 (5)	C5—C6—C1	117.8 (4)
Co1—O5—H5B	109.3	C5—C6—C8	113.7 (4)
Co1—O5—H5C	109.4	C1—C6—C8	128.5 (4)
H5B—O5—H5C	109.5	O1—C7—O2	120.9 (4)
Co1—O6—H6A	109.3	O1—C7—C1	118.8 (4)
Co1—O6—H6C	109.1	O2—C7—C1	120.3 (4)
H6A—O6—H6C	109.5	O3—C8—O4	121.2 (4)
Co1—O7—H7A	109.1	O3—C8—C6	118.9 (4)
Co1—O7—H7C	109.5	O4—C8—C6	119.9 (4)
H7A—O7—H7C	109.5		
			
C6—C1—C2—C3	0.9 (6)	C2—C1—C6—C8	179.1 (4)
C7—C1—C2—C3	−178.3 (4)	C7—C1—C6—C8	−1.9 (7)
C1—C2—C3—C4	1.4 (6)	C2—C1—C7—O1	−2.9 (6)
C1—C2—C3—C3^ii^	179.4 (4)	C6—C1—C7—O1	178.0 (4)
C2—C3—C4—C5	−2.8 (7)	C2—C1—C7—O2	175.6 (4)
C3^ii^—C3—C4—C5	179.2 (5)	C6—C1—C7—O2	−3.5 (7)
C3—C4—C5—C6	2.0 (7)	C5—C6—C8—O3	9.6 (6)
C4—C5—C6—C1	0.3 (7)	C1—C6—C8—O3	−171.2 (4)
C4—C5—C6—C8	179.6 (4)	C5—C6—C8—O4	−168.7 (4)
C2—C1—C6—C5	−1.8 (6)	C1—C6—C8—O4	10.4 (7)
C7—C1—C6—C5	177.3 (4)		

Symmetry codes: (i) −*x*+2, −*y*, −*z*+1; (ii) −*x*+2, −*y*+1, −*z*+2.

### Hydrogen-bond geometry (Å, °)

**Table:**

*D*—H···*A*	*D*—H	H···*A*	*D*···*A*	*D*—H···*A*
O2—H2···O4	0.85 (2)	1.55 (2)	2.391 (5)	173 (8)
O5—H5B···O4^iii^	0.96	2.17	2.820 (5)	124
O5—H5C···O2^iv^	0.96	1.97	2.789 (4)	142
O6—H6A···O3^v^	0.96	1.84	2.676 (4)	144
O6—H6C···O1^iv^	0.96	1.79	2.708 (4)	159
O7—H7A···O1^vi^	0.96	1.83	2.749 (5)	159
O7—H7C···O3	0.96	1.99	2.822 (5)	144

Symmetry codes: (iii) −*x*+1, −*y*, −*z*+1; (iv) *x*+1, *y*, *z*−1; (v) *x*+1, *y*, *z*; (vi) *x*, *y*, *z*−1.

## References

[bb1] Bruker (2001). *SADABS* Bruker AXS Inc., Madison, Wisconsin, USA.

[bb2] Bruker (2007). *APEX2* and *SAINT* Bruker AXS Inc., Madison, Wisconsin, USA.

[bb3] Kang, J., Huang, C.-C., Jiang, Z.-Q., Huang, S. & Huang, S.-L. (2009*a*). *Acta Cryst.* E**65**, m452. 10.1107/S1600536809008022 PMC2969031 21582388

[bb4] Kang, J., Huang, C.-C., Zhai, L.-S., Qin, X.-H. & Liu, Z.-Q. (2009*b*). *Acta Cryst.* E**65**, m380–m381. 10.1107/S1600536809007764 PMC2968794 21582332

[bb5] Sheldrick, G. M. (2008). *Acta Cryst.* A**64**, 112–122. 10.1107/S0108767307043930 18156677

[bb6] Sun, L.-X., Qi, Y., Che, Y.-X., Batten, S. R. & Zheng, J.-M. (2009). *Cryst. Growth Des.* **9**, 2995–2998.

[bb7] Wang, X.-L., Cao, Q. & Wang, E.-B. (2005). *Eur. J. Inorg. Chem.* pp. 3418–3421.

[bb8] Wang, X.-L., Cao, Q. & Wang, E.-B. (2006). *Cryst. Growth Des.* **6**, 439–433.

[bb9] Zhu, S., Zhang, H. & Shao, M. (2008). *Transition Met. Chem.* **33**, 669–680.

